# Association between the Serum Coenzyme Q10 Level and Seizure Control in Patients with Drug-Resistant Epilepsy

**DOI:** 10.3390/healthcare9091118

**Published:** 2021-08-28

**Authors:** Wei-Chen Liao, Chin-Wei Huang, Ya-Hsin Hsiao, Pi-Shan Sung, Tzu-Fun Fu, Alice Y. W. Chang, Hui Hua Chang

**Affiliations:** 1 Institute of Clinical Pharmacy and Pharmaceutical Sciences, College of Medicine, National Cheng Kung University, Tainan 701, Taiwan; weizhen60@gmail.com; 2 Department of Neurology, National Cheng Kung University Hospital, College of Medicine, National Cheng Kung University, Tainan 701, Taiwan; huangcw@mail.ncku.edu.tw (C.-W.H.); pishansung@gmail.com (P.-S.S.); 3 Institute of Basic Medical Sciences, College of Medicine, National Cheng Kung University, Tainan 701, Taiwan; yahsin@mail.ncku.edu.tw (Y.-H.H.); tffu@mail.ncku.edu.tw (T.-F.F.); aywchang@mail.ncku.edu.tw (A.Y.W.C.); 4 Institute of Behavioral Medicine, College of Medicine, National Cheng Kung University, Tainan 701, Taiwan; 5 Department of Pharmacology, College of Medicine, National Cheng Kung University, Tainan 701, Taiwan; 6 Department of Medical Laboratory Science and Biotechnology, College of Medicine, National Cheng Kung University, Tainan 701, Taiwan; 7 Department of Physiology, College of Medicine, National Cheng Kung University, Tainan 701, Taiwan; 8 School of Pharmacy, College of Medicine, National Cheng Kung University, Tainan 701, Taiwan; 9 Department of Pharmacy, National Cheng Kung University Hospital, College of Medicine, National Cheng Kung University, Tainan 701, Taiwan; 10 Department of Pharmacy, National Cheng Kung University Hospital, Dou-Liou Branch, Yunlin 640, Taiwan

**Keywords:** epilepsy, drug-resistant epilepsy, CoQ10, insulin, WHOQoL

## Abstract

Drug-resistant epilepsy (DRE) is a chronic neurological disorder with somatic impacts and increased risk of metabolic comorbidities. Oxidative stress might play an important role in metabolic effects and as a regulator of seizure control, while coenzyme Q10 (CoQ10) could improve insulin sensitivity through antioxidant effects. We aimed to investigate the association between CoQ10 level and clinical outcome, represented by the seizure frequency and quality of life, in DRE patients. DRE patients (*N* = 33) had significantly higher serum insulin levels and lower scores on the physical domain of the World Health Organization Quality of Life questionnaire (WHOQoL) than gender-age matched controls. The serum CoQ10 level (2910.4 ± 1163.7 ng/mL) was much higher in DRE patients than the normal range. Moreover, the serum CoQ10 level was significantly correlated with the seizure frequency (r = −0.412, *p* = 0.037) and insulin level (r = 0.409, *p* = 0.038). Based on stratification by insulin resistance (HOMA-IR > 2.4), the subgroup analysis showed that patients with a greater HOMA-IR had higher CoQ10 levels and lower seizure frequency, and had a significantly worse quality of life. In summary, CoQ10 could be a mediator involved in the mechanism of epilepsy and serve as a biomarker of the clinical outcome in DER patients.

## 1. Introduction

Epilepsy is a chronic neurological disorder caused by malfunctioning nerve cell activity in the brain, which is characterized by recurrent episodic attacks, epileptic seizures, and somatic impacts [1]. In addition, somatic conditions such as metabolic syndromes, arthritis, and heart diseases have also been linked to epilepsy [2,3,4]. The management of patients with epilepsy is focused on controlling seizures, avoiding treatment side effects, and restoring quality of life. Although there are expanding lists of available antiepileptic drugs (AEDs), approximately 30% of people who continue to have seizures after adequate trials of two AED treatments develop drug-resistant epilepsy (DRE) [5,6]. Patients with DRE have increased rates of medical and psychiatric comorbidities that could complicate epilepsy management, contribute to decreased health-related quality of life (HRQoL), increase health-care costs, and even shorten the lifespan [2,7]. Identifying the clinical features of DRE could have important implications for the development of new therapeutic approaches for epilepsy.

Recent reports have suggested that oxidative stress is involved in the pathophysiology of epilepsy [8]. Oxidative stress during and after seizures was instrumental in inducing immediate and long-term excitotoxic neuronal death, while inhibiting oxidative stress could have neuroprotective effects and modify the development of epilepsy [8]. Thus, coenzyme CoQ10 (CoQ10) might play a key role in epilepsy [9,10]. Coenzyme Q (ubiquinone), an endogenously synthetized redox-active lipid, is considered to be an electron carrier of the mitochondrial respiratory chain with antioxidant properties [11]. In humans, coenzyme Q, which contains 10 isoprene units, can be found in most endomembranes such as the plasma membrane and serum lipoproteins, and is abundant in mitochondria [12]. CoQ10 levels decline with aging, which might be due to decreased synthesis or an increase in oxidative damage [13]. CoQ10 deficiency could lead to insulin resistance and neurodegenerative disorders such as Alzheimer’s disease and Parkinson’s disease [11,14]. Previous reports have shown that CoQ10 could improve glycemic control in diabetes mellitus (DM) patients by improving insulin secretion and reducing oxidative stress [15,16]. Moreover, CoQ10 supplementation with AED could attenuate seizures in pilocarpine-induced seizure animal model studies [9,10].

Oxidative stress might also contribute to the high comorbidities of metabolic disturbances in patients with epilepsy [17,18]. Seizures result in the alteration of glucose metabolism, the reduction of intracellular energy metabolites, and the accumulation of metabolic intermediates, such as lactate and adenosine [18,19]. Furthermore, although studies have reported that glucose dysregulation may be a predictor of the pathological outcome [20,21], little is known about the effect of glycemic control on clinical outcome in patients with DRE. In addition, CoQ10 can serve as a regulator of the insulin and adiponectin receptors, tyrosine kinase, phosphatidylinositol kinase, and glucose transporters, which suggests that its antioxidant effect improves insulin sensitivity [22,23,24]. Therefore, oxidative stress may not only possess metabolic effects but may also play a role as a regulator of seizure control. Thus, we aimed to investigate the association between oxidative stress, the CoQ10 level, and clinical outcome in patients with DRE. Additionally, it would be of interest to investigate whether insulin sensitivity stratifies the clinical outcomes in patients with DRE. The clinical outcome was represented by seizure frequency and HRQoL in the current study.

## 2. Materials and Methods

### 2.1. Subjects

The research protocol was approved by the Ethical Committee for Human Research at the National Cheng Kung University (NCKU) Hospital (IRB No. A-ER-105–489), and written informed consent was obtained from each subject before any procedures were performed. This study was conducted in accordance with the Declaration of Helsinki. The participants (aged 20–65 years) were enrolled consecutively by a trained neurologist and diagnosed with refractory epilepsy. The inclusion criteria for refractory epilepsy were (1) a diagnosis of epilepsy, and (2) the failure of two or more antiepileptic drugs (AEDs) and the occurrence of one or more seizures per month over 18 months [25,26]. Participants undergoing surgery for epilepsy, with an organic mental disorder, mental retardation, dementia, or other diagnosed neurological illness, with a surgical condition or a major physical illness, or who were pregnant or breastfeeding, were excluded from our study. The seizure frequency was defined as the number of episodes per 28 days based on medical records from the neurologists and was used to assess the severity of the disease. To investigate the quality of life in patients with epilepsy, we also recruited controls from the community through an advertisement, which recruited subjects who were without neurological illness, psychiatric illness, severe physical illness (such as cardiovascular diseases and cancers), or a past history of inflammatory diseases (such as type 2 diabetes mellitus and hyperlipidemia). In addition, the body weight and height of each subject were measured, and the BMI (kg/m^2^) was calculated accordingly.

### 2.2. Blood Sample Collection

Blood samples were collected from the antecubital vein in heparinized plain tubes after fasting for 8 to 12 h. Serum and plasma were separately isolated from whole blood by centrifugation at 1500 rpm for 15 min at 4 °C and were then immediately stored at −80 °C. 

### 2.3. Creatine Phosphokinase (CPK) Level

Plasma CPK levels were detected by an automatic dry biochemical analyzer (FUJI DRI-CHEM 4000 i). The detection range was 10–2000 IU/L.

### 2.4. Fasting Sugar Profiles

Fasting plasma glucose values were determined using the glucose oxidase method (Synchron CX3; Beckman, Chaska, MN, USA), and the fasting serum insulin concentrations were measured by a solid-phase radioimmunoassay method (Diagnostic Products Corporation, Los Angeles, CA, USA). HbA1c and fasting serum insulin concentrations were measured by a solid-phase radioimmunoassay method (Diagnostic Products Corporation, Los Angeles, CA, USA). All blood sugar profiles were measured at the laboratory of the Pathology Research Center at NCKU Hospital. Finally, insulin resistance conditions and pancreatic β-cell function were estimated using the homeostasis model assessment-estimated insulin resistance (Homeostatic model assessment for insulin resistance, HOMA-IR) index and the homeostasis model assessment of pancreatic β-cell function (Homeostatic model assessment for β-cell function, HOMA-β) [27]. The McAuley Index was used for predicting insulin resistance in normoglycemic individuals [28].

### 2.5. Serum Vitamin CoQ10 Level

Serum CoQ10 level was measured with a CoQ10 ELISA Kit (MyBioSource, Inc., San Diego, CA, USA). The limit of detection was 0.781 ng/mL, and the intra and interassay coefficients of variation (CV) were less than 8% and 10%, respectively.

### 2.6. World Health Organization Quality of Life (WHOQoL)

The Taiwanese version of the World Health Organization Quality of Life-BREF (WHOQoL-BREF) was used to measure the overall and specific quality of life in all subjects [29]. This questionnaire consists of 28 items in four domains: physical, psychological, social relations and environment. The reliability and validity of the Taiwanese version of the WHOQoL-BREF were tested. The test–retest reliability coefficient at intervals of 2 to 4 weeks ranged from 0.76 to 0.80 at the domain level. The internal consistency (Cronbach’s alpha) coefficients were in the range of 0.70 to 0.77 for the four domains, and the content validity coefficients were in the range of 0.53 to 0.78 for item-domain correlations.

### 2.7. Statistical Analysis

We analyzed data with the Statistical Package for Social Sciences version 18.0 (SPSS Inc., Chicago, IL, USA). All demographic and clinical characteristics of the subjects were calculated either as numbers and percentages for categorical variables or as the mean ± standard deviation for continuous variables. Student’s *t*-test and chi-square (χ^2^) tests were used to evaluate the differences in the characteristics between groups. Associations were tested by Spearman’s correlation test due to the result of the normality test for variables. All tests were two-sided, and a *p* value < 0.05 was considered statistically significant unless otherwise specified.

## 3. Results

We recruited 33 DRE patients and 33 age and gender-matched controls (66.7% of females). Age, gender, BMI and the fasting glucose level did not significantly differ between the DRE patients and the controls. However, the serum levels of insulin (9.46 ± 5.50 vs. 6.60 ± 3.39 µIU/mL, *p* = 0.014), HOMA-IR (2.12 ± 1.28 vs. 1.47 ± 0.80, *p =* 0.016), and HOMA-β (143.24 ± 84.14 vs. 92.54 ± 46.07%, *p* = 0.004) were higher in DRE patients than those in the controls, while the HbA1c was lower (5.25 ± 0.60 vs. 5.59 ± 0.31%, *p* = 0.007) (Table 1). In addition, the serum CoQ10 level (2910.4 ± 1163.7 ng/mL) was much higher in DRE patients than the normal range in the general population (360~1590 ng/mL) [30], while the CPK level was within the normal range (Table 1). Furthermore, DRE patients had lower scores in the physical domain of the WHOQoL questionnaire than controls (12.94 ± 2.42 vs. 14.56 ± 1.62, *p* = 0.002) (Table 1).

Since the serum CoQ10 level was significantly increased in patients, we further investigated whether the serum CoQ10 level was correlated with the clinical features of DRE patients. The results showed that the serum CoQ10 level was significantly correlated with seizure frequency (r = −0.412, *p* = 0.037) and insulin (r = 0.409, *p* = 0.038) (Table 2). In addition, we investigated the correlation of CPK levels and clinical characteristics. The results showed that the CPK level was significantly correlated with seizure frequency (r = −0.385, *p* = 0.039), HOMA-β (r = 0.391, *p* = 0.033), and the McAuley index (r = 0.382, *p* = 0.041) (Table 2).

Considering that the insulin level could stratify the DRE patients and controls, we further subgrouped patients according to a cutoff value for the HOMA-IR ≥ 2.4 [31,32,33], which represents the status of insulin resistance (Table 3). The results showed that patients with a HOMA-IR greater than 2.4 had a significantly increased BMI (27.04 ± 5.73 vs. 21.84 ± 2.79, *p* = 0.038), insulin level (16.23 ± 6.97 vs. 7.20 ± 2.18 µIU/mL, *p* = 0.008), and HOMA-IR (3.80 ± 1.43 vs. 1.56 ± 0.53, *p* = 0.003) and a decreased McAuley Index (5.57 ± 0.68 vs. 9.07 ± 1.87, *p* < 0.001). Interestingly, these patients also had significantly increased serum CoQ10 levels (4387.00 ± 1351.67 vs. 2612.12 ± 826.84 ng/mL, *p* = 0.001) and tended to have a decreased seizure frequency (1.71 ± 1.31 vs. 8.58 ± 7.88, *p* = 0.074). However, patients with a HOMA-IR>2.4 had significantly lower WHOQoL scores in the physical domain (11.21 ± 2.26 vs. 13.29 ± 2.05, *p* = 0.022) and total scores (86.18 ± 10.69 vs. 96.25 ± 12.18, *p* = 0.046) than patients with a HOMA-IR ≤ 2.4.

## 4. Discussion

To our knowledge, this is the first clinical study to identify the association between CoQ10 level and clinical outcome in DRE patients. We found that DRE patients had increased CoQ10 levels and insulin profiles and a worse health-related quality of life than the controls. Moreover, DRE patients with increased CoQ10 levels had a decreased seizure frequency and increased insulin levels. The results suggest that the balancing of CoQ10 levels might be critical for seizure control as well as insulin-related metabolic syndrome in refractory seizure cases. Therefore, CoQ10 might not only be a mediator involved in the mechanism of disease but also a biomarker of the clinical outcome in DER patients.

CoQ10 is an antioxidant compound [30] that exhibits some therapeutic and neuroprotective effects due to its potent antioxidant capacity. CoQ10 has been found to be a safe and effective adjuvant for AED therapy in epilepsy rodent models, suggesting that it could ameliorate seizure severity and protect against seizure-induced oxidative damage causing cognitive impairment associated with chronic use of AEDs [10,34]. The possible underlying molecular mechanism by which CoQ10 alleviates phenytoin-induced cognitive impairment is related to VEGF and enhances the BDNF-TrkB-CREB signaling pathway in the hippocampus and cortex of phenytoin-treated rats [35]. In addition, CoQ10 showed antiseizure activity through the induction of constitutive nitric oxide synthase expression in pentylenetetrazole or electroshock-induced mouse models [9]. Similar to previous reports from animal studies, our clinical study demonstrated that DRE patients with increased CoQ10 levels had decreased seizure frequency. However, whether CoQ10 supplementation could prevent or rescue cognitive impairment is still unclear in patients with epilepsy. Taken together, the results suggest that the usefulness of CoQ10 as an adjuvant for AEDs could play an important role in preventing cognitive impairment and oxidative stress in epilepsy. Further studies are needed to clarify the mechanism of CoQ10 supplementation and to confirm its effect on cognitive function in patients with epilepsy.

In addition, both energy homeostasis and oxidative stress might share a common pathway in epilepsy, since our study found that the CoQ10 level was significantly positively correlated with insulin level. Previous reports indicated that seizures result in altered glucose metabolism, a reduction in intracellular energy metabolites and the accumulation of metabolic intermediates, such as lactate. Hyper or hypoglycemia additively increased the extent of seizure-induced cell death in an excitotoxin-resistant mouse study [20]. Moreover, the duration and extent of glucose dysregulation are suggested to be predictors of the pathological outcome in patients with epilepsy. Thus, the homeostasis of insulin signaling represents a critical factor and is an important therapeutic target. In addition, AEDs could result in the alteration of insulin homeostasis in patients with epilepsy [36,37]. Treatment with valproic acid, a broad-spectrum antiepileptic drug used for therapy of generalized and focal epilepsies, is associated with obesity and hyperinsulinemia. It is involved in dysregulation of the hypothalamic system and adipokine levels and genetic susceptibility [38]. Compared with valproic acid, carbamazepine and topiramate have a reduced risk of altering insulin homeostasis and causing obesity [38,39]. Moreover, although levetiracetam, which is the most commonly used drug in our DRE patients, is considered weight-neutral, its effects on insulin homeostasis are still unclear [40]. In our current study, patients with an increased HOMA-IR had a decreased seizure frequency but worse quality of life. Whether the dysregulation of insulin levels is influenced by epilepsy itself, the types of AEDs used, or both, has not been fully studied. Moreover, a positive correlation between insulin and CoQ10 levels was also noted in our study. Taken together, the results indicate that an improved understanding of the possible shared mechanism involved in epilepsy, glucose metabolism, insulin signaling, and the actions of AEDs could contribute to the design of therapies targeting molecules besides those targeted by typical ion channel AEDs. Alternatives to current drug therapies, such as a ketogenic diet and vitamin supplementation, have shown effectiveness and beneficial cellular effects [17,41,42]. Although metabolic disturbances are often associated with epilepsy and AEDs, little is known about either the effects of glycemic control on brain metabolism or the effects of managing systemic glucose concentrations in epilepsy. The pathogenesis and mechanism remain to be further studied.

There were some limitations of the present study. The first limitation was that the sample size in this single-site study was relatively small because of the low prevalence of refractory epilepsy. The second limitation was the lack of profiles of the CoQ10 level and CPK level for the controls in the current study. The third limitation was that the CoQ10 level was measured in the peripheral nervous system but not in the central nervous system. The fourth limitation was that we did not correct the multiple comparisons in the correlations between serum CoQ10 and clinical characteristics, in which alpha-errors could be accumulated. The fifth limitation was that since we focused on DRE patients in the current study, the findings could not be extrapolated to drug-sensitive patients. In addition, we did not recruit drug-sensitive patients so we could not conclude that the findings are only specific to DRE patients. A longitudinal study with a large sample size is necessary to elucidate the serum CoQ10 and treatment outcomes in patients with epilepsy.

## 5. Conclusions

In summary, the study demonstrated that DRE patients had increases in CoQ10 levels and insulin profiles and a worse health-related quality of life than controls. DRE patients with increased CoQ10 levels had decreased seizure frequencies and increased insulin levels. Therefore, the balance of CoQ10 levels plays a crucial role in seizure control as well as insulin-related metabolic syndrome in refractory seizure cases. CoQ10 and insulin might be mediators involved in the mechanism and biomarkers of clinical outcome in DER patients. Since identifying the clinical features of DRE has important implications for the development of new therapeutic approaches, and contributes to the accuracy of the prediction of the AEDs response, the mechanism of CoQ10-related seizure control is also pertinent for further investigations.

## Figures and Tables

**Table 1:** Demographic characteristics of the AED-resistant epilepsy patients and the control subjects (1:1 matched age and gender).

	AED-Resistant EP ^a^ (*n* = 33)	Control (*n* = 33)	Comparison			
Characteristics	Mean ± SD	Mean ± SD	t/χ^2^	95% CI		*p*-Value
				Lower	Upper	
Gender (male, %)	11 (33.3)	11 (33.3)	0.00	-	-	1.000
Age, years	41.42 ± 10.22	40.10 ± 10.32	0.52	−3.73	6.37	0.603
BMI, kg/m^2^	23.14 ± 4.30	23.90 ± 3.66	−0.77	−2.73	1.22	0.445
CoQ10, ng/mL	2910.4 ± 1163.7	(normal range: 360~1590)				
CPK, IU/L	125.53 ± 60.39	(normal range: 30~223)				
Blood sugar profile						
AC glucose, mg/dL	89.81 ± 16.08	88.88 ± 6.73	0.31	−5.14	7.01	0.760
Insulin, uIU/mL	9.46 ± 5.50	6.60 ± 3.39	2.53	0.60	5.12	0.014 *
HOMA-IR	2.12 ± 1.28	1.47 ± 0.80	2.47	0.13	1.18	0.016 *
HOMA-Beta, %	143.24 ± 84.14	92.54 ± 46.07	3.03	17.22	84.19	0.004 *
HbA1c, %	5.25 ± 0.60	5.59 ± 0.31	−2.82	−0.58	−0.10	0.007 *
McAuley Index	8.28 ± 2.23	8.92 ± 1.87	−1.21	−1.71	0.42	0.233
WHOQoL score						
Physical domain	12.94 ± 2.42	14.56 ± 1.62	−3.21	−2.64	−0.61	0.002 *
Psychological domain	13.52 ± 2.20	13.56 ± 1.49	−0.09	−0.97	0.89	0.931
Social domain	13.91 ± 2.48	13.88 ± 1.76	0.06	−1.03	1.09	0.955
Environment domain	14.11 ± 2.14	14.09 ± 1.69	0.06	−0.92	0.98	0.955
Integrated QOL	3.27 ± 0.94	3.27 ± 0.57	0.00	−0.39	0.39	1.000
Integrated Health	2.94 ± 0.79	3.03 ± 0.30	−0.62	−0.39	0.21	0.540
Total scores	93.73 ± 12.47	97.70 ± 8.94	−1.48	−9.33	1.40	0.145

Data are presented as mean ± SD or number (percentage). Abbreviations: AED, antiepilepsy drug; BMI, body mass index; AC glucose, Glucose Ante Cibum; HOMA-IR, Homeostatic Model Assessment for Insulin Resistance; HOMA-Beta, Homeostasis Model Assessment of β-cell Function; HbA1c, Glycated hemoglobin A1c; WHOQoL, World Health Organization Quality of Life questionnaire. All the reverse questions of WHOQoL were conversed, and score were adjusted in the range 4 to 20 (Higher scores represent a better condition). a: The inclusion criteria for the AED-resistant EP group met the following criteria: (1) aged >20 years, (2) diagnosis of refractory epilepsy. * *p* < 0.05.

**Table 2:** The correlation between serum CoQ10 level, CPK level and other characteristics of AED-resistant epilepsy patients.

Parameters	CoQ10		CPK	
	σ	Spearman’s *p*	σ	Spearman’s *p*
Seizure frequency, episode/28 days	−0.412	0.037 *	−0.385	0.039 *
BMI, kg/m^2^	0.193	0.344	0.207	0.273
CPK, IU/L	0.231	0.257	-	-
Blood sugar profile				
AC glucose, mg/dL	0.013	0.949	0.117	0.538
Insulin, µIU/mL	0.409	0.038 *	−0.322	0.082
HOMA-IR	0.378	0.057	−0.242	0.197
HOMA-Beta, %	0.299	0.138	−0.391	0.033 *
HbA1c, %	−0.168	0.412	0.014	0.940
McAuley Index	−0.368	0.065	0.382	0.041 *

Correlations between baseline serum CoQ10 levels in AED-resistant epilepsy patients were analyzed by Spearman’s correlation. Abbreviations: BMI, body mass index HOMA-IR, Homeostatic Model Assessment for Insulin Resistance; HOMA-Beta, Homeostasis Model Assessment of β-cell Function; HbA1c, Glycated hemoglobin A1c; CPK, Human creatine phosphokinase. * *p* < 0.05.

**Table 3:** Demographic characteristics of AED-resistant epilepsy patients subgrouped by HOMA-IR.

	HOMA-IR > 2.4 (*n* = 9)	HOMA-IR ≤ 2.4 (*n* = 24)	Comparison			
Baseline Characteristics	Mean ± SD	Mean ± SD	t/χ^2^	95% CI		*p*-Value
				Lower	Upper	
Gender (male, %)	2 (25.0)	9 (37.5)	0.42			0.519
Age, years	41.56 ± 11.24	41.88 ± 9.50	−0.08	−7.83	7.20	0.933
Seizure frequency, episode/28 days	1.71 ± 1.31	8.58 ± 7.88	−1.87	−14.47	0.72	0.074
BMI, kg/m^2^	27.04 ± 5.73	21.84 ± 2.79	2.47	0.36	10.04	0.038 *
CoQ10, ng/mL	4387.00 ± 1351.67	2612.12 ± 826.84	3.82	814.62	2735.15	0.001 *
CPK, IU/L	108.33 ± 41.18	129.83 ± 64.30	−0.78	−78.36	35.36	0.445
Blood sugar profile						
AC glucose, mg/dL	98.38 ± 27.39	86.96 ± 9.29	1.16	−11.58	34.41	0.283
Insulin, µIU/mL	16.23 ± 6.97	7.20 ± 2.18	3.60	3.18	14.88	0.008 *
HOMA-IR	3.80 ± 1.43	1.56 ± 0.53	4.31	1.03	3.44	0.003 *
HOMA-Beta, %	222.22 ± 130.59	116.91 ± 38.21	2.25	−4.20	214.82	0.057
HbA1c, %	5.57 ± 0.79	5.15 ± 0.50	1.81	−0.06	0.91	0.081
McAuley Index	5.57 ± 0.68	9.07 ± 1.87	−7.62	−4.45	−2.56	<0.001 *
WHOQoL score						
Physical domain	11.21 ± 2.26	13.29 ± 2.05	−2.42	−3.83	−0.32	0.022 *
Psychological domain	12.33 ± 1.82	13.67 ± 1.94	−1.71	−2.93	0.26	0.097
Social domain	12.67 ± 2.21	14.07 ± 2.21	−1.55	−3.25	0.44	0.131
Environment domain	13.11 ± 1.94	14.22 ± 1.89	−1.43	−2.70	0.48	0.163
Integrated QOL	3.00 ± 1.20	3.29 ± 0.81	−0.78	−1.05	0.47	0.440
Integrated Health	2.88 ± 1.13	2.96 ± 0.69	−0.25	−0.76	0.60	0.803
Total scores	86.18 ± 10.69	96.25 ± 12.18	−2.08	−19.95	−0.19	0.046 *

Data are presented as mean ± *SD* or number (percentage). Abbreviations: AED, antiepilepsy drug; BMI, body mass index; AC glucose, Glucose Ante Cibum; HOMA-IR, Homeostatic Model Assessment for Insulin Resistance; HOMA-Beta, Homeostasis Model Assessment of β-cell Function; HbA1c, Glycated hemoglobin A1c; WHOQoL, World Health Organization Quality of Life questionnaire. All the reverse questions of WHOQoL were conversed, and score were adjusted in the range 4 to 20 (Higher scores represent a better condition). * *p* < 0.05.

## Data Availability

The data that support the findings of this study are available from National Cheng Kung University. Restrictions apply to the availability of these data, which were used under license for this study. Data are available for Hui Hua Chang with the permission of National Cheng Kung University.
